# Hippocampal AMPARs involve the central sensitization of rats with irritable bowel syndrome

**DOI:** 10.1002/brb3.650

**Published:** 2017-02-22

**Authors:** Aiqin Chen, Yu Chen, Ying Tang, Chengjia Bao, Zizhi Cui, Meng Xiao, Chun Lin

**Affiliations:** ^1^ Fujian Provincial Key Laboratory of Neuroglia and DiseasesLaboratory of Pain ResearchDepartment of Physiology and PathophysiologyFujian Medical UniversityFuzhouFujianChina; ^2^ 2013 Seven‐year Clinical MedicineFujian Medical UniversityFuzhouFujianChina

**Keywords:** AMPARs, hippocampus, irritable bowel syndrome, long‐term potentiation, visceral hypersensitivity

## Abstract

**Objective:**

The roles of hippocampal AMPARs were investigated in irritable bowel syndrome (IBS)‐like rats to clarify the central sensitization mechanisms.

**Methods:**

IBS model was induced by neonatal maternal separation. The effects of AMPARs on visceral hypersensitivity were examined by the responses of abdominal muscle to colorectal distension after the bilateral intrahippocampal injections of CNQX (an AMPAR inhibitor). The expressions of hippocampal AMPARs (GluR1 and GluR2) were determined by Western blot.

**Results:**

The IBS‐like rats showed visceral hypersensitivity when compared with controls. Bilateral intrahippocampal injections of CNQX alleviated the visceral pain in IBS‐like rats. The maximal effect appeared at the time point of 30 min, and the duration lasted for 90 min after CNQX application, under 40 and 60 mmHg CRD. The expressions of hippocampal GluR2 significantly increased in IBS‐like rats when compared with controls (*p *<* *.05). However, the levels of hippocampal GluR1 had no significant differences in rats. Hippocampal LTP induced by HFS was significantly enhanced when compared with controls (*p *<* *.05). The expressions of GluR2 significantly increased in the control and IBS‐like rats after 60 min LTP of recordings (*p *<* *.05), but not GluR1.

**Conclusion:**

Neonatal maternal separation enhances the expression of GluR2 and facilitates the LTP in the hippocampus, which could lead to the formation of visceral hypersensitivity when grown up.

## 1 Introduction

Irritable bowel syndrome (IBS) influences around 11% of the population globally (Canavan, West, & Card, 2014). It manifests as abdominal hypersensitivity and abnormal gastrointestinal function. The IBS patients suffer huge psychological burdens and usually need more medications. IBS turns out to be a great social and economic burden (Plavsic, Hauser, Tkalcic, Pletikosic, & Salkic, 2015). Thus, greater efforts have been made to explore the mechanism of IBS and also in search of new therapies for it.

It is widely accepted that visceral hypersensitivity is the main factor causing visceral pain and bowel motor disorders in IBS patients (Agrawal et al., 2008). Visceral hypersensitivity results from peripheral sensitization and central sensitization (Price, Zhou, Moshiree, Robinson, & Verne, 2006): the former refers to sensitization of primary noxious sensor, and the latter indicates a persistent central synaptic transmission change during injury or after injury. Previously, most researches about the mechanism of visceral hypersensitivity focused on peripheral sensitization and the synaptic plasticity change in cornu dorsal medullae spinalis. Little attention has been paid to higher central synaptic plasticity.

Pain is conducted by a complex neural network in the central nervous system (Liu & Chen, 2009). Chronic pain not only manifests hyperalgesia, allodynia, and spontaneous pain but also leads to various mood diseases, such as anxiety and depression (Liu & Chen, 2009). Thus, studies concerning pain are no longer restricted to the spinal cord, anterior cingulate, and almond, which have long been accepted as pain centers. The hippocampus also participates in pain and painful memories (Currie & Wang, 2004; Minami, 2012). Chronic pain lasts after the damage has cured. It is because of the functional or structural changes in the brain like memory processes. Therefore, chronic pain has been considered to be “a persistence of pain memory and the inability to extinguish the memory of pain evoked by an initial inciting injury”(Apkarian, Baliki, & Geha, 2009). A greater number of researchers believe that chronic pain shares a similar mechanism with memory, i.e., long‐term potentiation. The hippocampus plays a key role in memory; thus, we choose it as the target of our study.

A huge number of experimental results have revealed that glutamate is an excitatory neurotransmitter that mediates painful information transfer. The a‐amino‐3‐hydroxy‐ 5‐methyl‐4‐isoxazole‐propionate receptors (AMPARs) are glutamate receptors widespread in the brain. There are four subunits in most AMPARs: GluR1, GluR2, GluR3, and GluR4 (Song & Huganir, 2002). GluR1 and GluR2 are highly expressed in the I‐II layers of the dorsal horn, where noxious inputs end, but GluR3 and GluR4 are sparsely expressed (Kopach & Voitenko, 2013). Our previous results suggest that spinal AMPARs may participate in the process of central hypersensitivity (Lin & Al‐Chaer, 2005), but the roles of hippocampal AMPARs in chronic visceral pain remain unclear.

In this study, an IBS model was induced by neonatal maternal separation as in our previous studies. The effects of intrahippocampal injections of CNQX (an AMPAR inhibitor) on visceral hypersensitivity were examined using electromyogram (EMG), i.e., the responses of abdominal muscle to colorectal distension (CRD). The expressions of hippocampal AMPARs (GluR1 and GluR2) were evaluated by Western blot. In addition, the field potentials of CA1 region were recorded in the slices to figure out the hippocampal LTP. Finally, the expressions of GluR1 and GluR2 in the slices were evaluated by Western blot 60 min after high‐frequency stimulation (HFS). A plausible mechanism of central hypersensitivity of chronic visceral pain in IBS was raised.

## 2 Material and Methods

### 2.1 Animals

Sprague‐Dawley (SD) rats were obtained from the Experimental Animal Center of Fujian Medical University. Male rats (about 250 g) were used in the following study when they were 8 weeks old. IBS‐like rats were induced by neonatal maternal separation (NMS) for three hours a day during postnatal days 3–21 (Chen et al., 2014; Xiao et al., 2016). The controls were treated just like the IBS‐like rats except for NMS. Rats have free access to food and water. They were maintained on a 12‐hours light/dark cycle. All procedures were conducted during the light cycle. The rats were monitored routinely at least once daily by researchers. Rats were gently handled before experiments to alleviate stress and anesthetized during recording. Euthanasia was performed by administering an intraperitoneal injection of a lethal dose of pentobarbital sodium (150–200 mg/kg) at the end of the experiments. All animal procedures were approved by the Committee for Care and Use of Laboratory Animals at Fujian Medical University.

### 2.2 Measurement of visceral sensitivity

The visceral hypersensitivity was assessed as described before (Chen et al., 2014) by recording the spikes of the abdominal muscle to CRD (40 mmHg, 60 mmHg) when they were 8 weeks old. Distention balloons were placed in the descending colons under isoflurane anesthesia (VMR, Matrix, USA). Then, silver bipolar electrodes were inserted into the abdominal muscle. Distention was produced by rapidly inflating the balloon (10 s every 4 min) to the desired pressure. The average of three recordings is taken as the amplitude of EMG. The EMG recordings were collected and analyzed using RM6240BD (Chengyi, China).

### 2.3 Surgery and intrahippocampal injection

After anesthesia, rats were placed in a stereotaxic instrument (Narishige, Japan). Through a midline incision along the skull, a stainless steel guide cannula was put stereotaxically and directed into the CA1 region of the hippocampus (Chen et al., 2014). Dental resin was used to fix two cannulae on the skull with two little screws. After a 3‐day recovery period, 2 μl CNQX was injected into the hippocampal CA1 region within 5 min. DMSO was injected to the controls in the same way.

### 2.4 Hippocampal electrophysiological recordings

The Schaffer collateral‐Commissural pathway was stimulated and fEPSPs were recorded from the dendritic layer of the CA1 pyramidal cells as reported by Kleppisch (Chen et al., 2014; Kleppisch et al., 1999). In the experiments, the amplitudes of the fEPSPs of the downward peak were measured. The test stimulating frequency was 0.1 Hz. The intensity was adjusted to produce about 50% of the maximal field potential amplitude. The baseline field potential was recorded at least 10 min before LTP induction or administration of agents. LTP was induced by HFS and recorded 60 min after HFS (Chen et al., 2014). The fEPSPs were amplified and analyzed using the RM6240BD.

### 2.5 Western blotting

The expressions of GluRs were assessed as described by Luo et al. (2014). 30 μg proteins from the hippocampus were extracted and transferred onto PVDF membranes (Invitrogen, USA), which were probed with rabbit anti‐GluR1 polyclonal antibody (1:1000; Millipore, Cat#AB1504, RRID:AB_2113602), mouse anti‐GluR2 monoclonal antibody (1:1000; Millipore, Cat#MAB397, RRID:AB_2113875) and mouse anti‐β‐actin monoclonal antibody(1:1000; EarthOx Life Sciences Cat# E021020, RRID:AB_2572416). Blots were rinsed, followed by incubation in peroxidase‐conjugated goat anti‐rabbit IgG (1:10000; Beijing Emarbio Science & Technology Co., Ltd Cat# EM35111, RRID:AB_2572420) or peroxidase‐conjugated goat anti‐mouse Ig G (1:10000; EarthOx Life Sciences Cat# E030110, RRID:AB_2572419). An ECL system (Xiamen Lulong Biotech Co., Ltd.) was used to visualize the bands.

### 2.6 Statistical analysis

SPSS 10.0 was used to perform the analysis. A one‐way repeated measures analysis of variance (ANOVA) was used to compare the EMG between control and IBS‐like rats. One or two‐way repeated measures ANOVA and Bonferroni post hoc test were adopted for multiple comparisons after the intrahippocampal injection of compounds. Western blot results were examined using unpaired *t*‐test. The student's t‐test was used to compare the hippocampal field potential in control and IBS‐like rats. One‐way ANOVA with Student–Newman–Keuls (SNK) multiple comparisons post‐hoc analysis was used to compare the different groups of hippocampal slices of rats in vitro. All data were expressed as mean ± SEM. *p *< .05 was considered to be statistically significant.

## 3 Results

**Figure 1:**
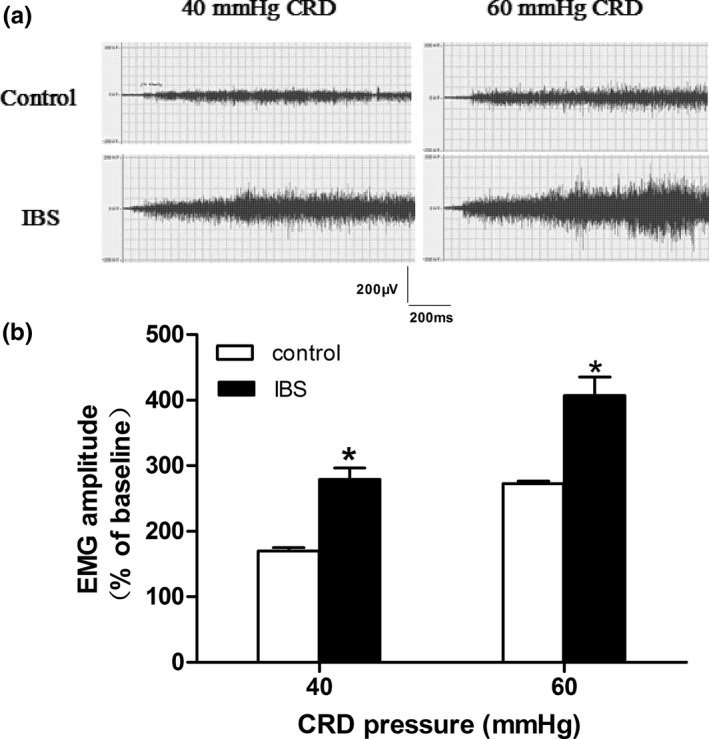
EMG recordings of abdominal muscle to CRD in control and IBS‐like rats. (a) The original typical recordings of EMG under 40, 60 mmHg CRD pressure in control and IBS‐like rats. (b) The statistical chart of the percentage of EMG amplitude over baseline. The formula of calculating EMG to CRD is equal to (CRD responses‐baseline)/baseline × 100%. *n *= 7, *: *p *<* *.05, vs controls. EMG: electromyography; CRD: colorectal distension; IBS: irritable bowel syndrome

**Figure 2:**
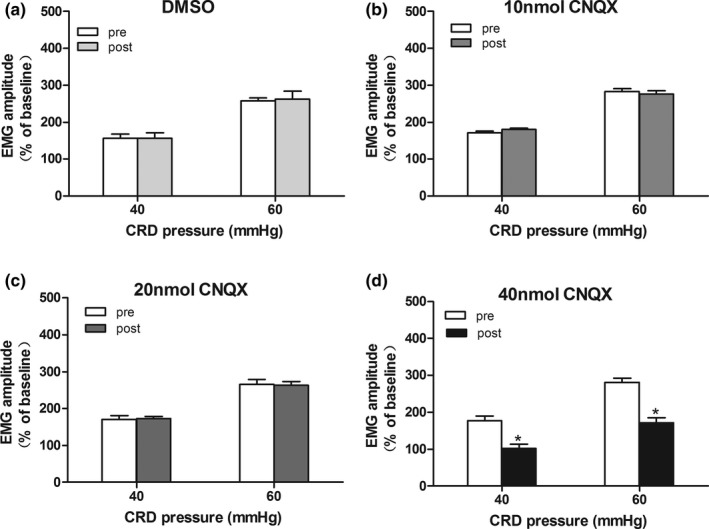
Effects of CNQX on visceral hypersensitivity in control rats. The statistical chart of EMG amplitude to CRD (40, 60 mmHg) after intrahippocampal injections of DMSO (a), 10 nmol CNQX (b), 20 nmol CNQX (c) and 40 nmol CNQX (d) in controls. *n *= 7, *: *p *<* *.05, vs. pre‐drug. EMG, electromyography; CRD, colorectal distention; IBS, irritable bowel syndrome; CNQX, an AMPAR inhibitor

**Figure 3:**
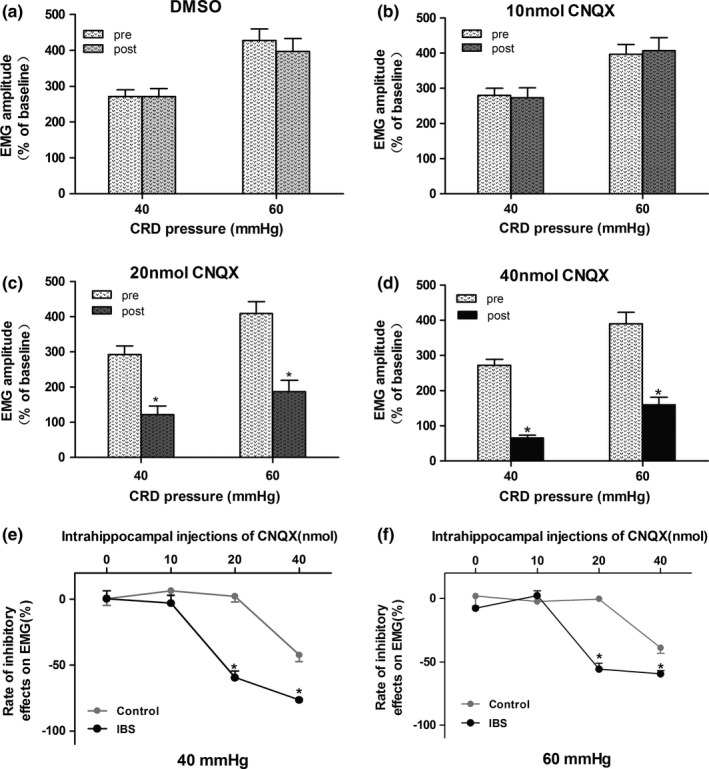
Effects of CNQX on visceral hypersensitivity in rats. (a–d) The statistical chart of EMG amplitude to CRD (40, 60 mmHg) after intrahippocampal injections of DMSO and 10–40 nmol CNQX in IBS‐like rats. (e, f) The inhibitory rate of 10–40 nmol CNQX on visceral hypersensitivity under 40 and 60 mmHg CRD in control and IBS‐like rats. *n *= 7, *: *p *<* *.05, vs. pre‐drug. EMG: electromyography; CRD, colorectal distention; IBS, irritable bowel syndrome; CNQX, an AMPAR inhibitor

**Figure 4:**
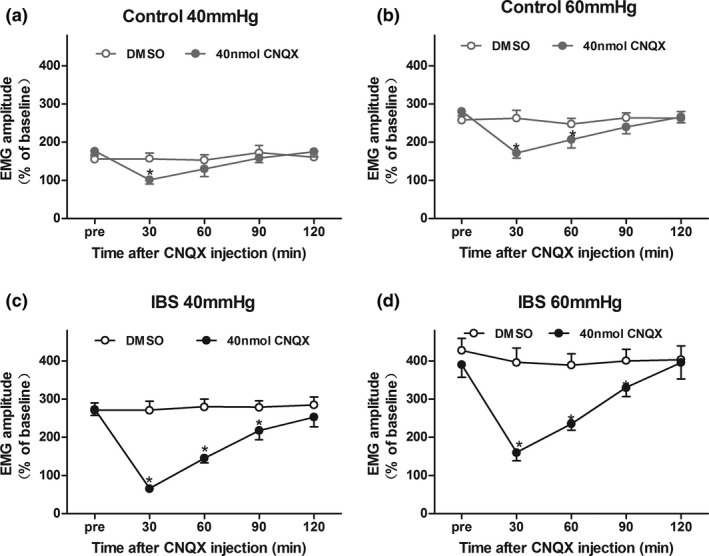
The time curve of CNQX effects on visceral hypersensitivity in rats. The time curve of the inhibitory effects of 40 nmol CNQX on EMG under 40 and 60 mmHg CRD in control (a, b) and IBS‐like rats (c, d). The maximal inhibition was observed at 30 min after CNQX application in control and IBS‐like rats. The inhibitory effects of CNQX lasted for 90 min in IBS‐like rats, but 30/60 min in control rats. *n *= 7, *:*p *<* *.05, vs pre‐drug. EMG, electromyography; CRD, colorectal distention; IBS, irritable bowel syndrome; CNQX, an AMPAR inhibitor

IBS‐like rats had visceral hypersensitivity when compared with controls (Figure 1, *p *< .05). Bilateral intrahippocampal injections of 40 nmol CNQX attenuated the visceral hypersensitivity in controls (Figure 2d, *p *< .05). However, no significant alleviation was found between the DMSO groups with either 10 or 20 nmol CNQX (Figure 2a–c). Bilateral intrahippocampal injections of CNQX has dose‐dependently decreased the visceral pain sensitivity in IBS‐like rats (Figure 3, *p *< .05). The greatest alleviation was recorded at the time point of 30 min, and the duration lasted for 90 min after CNQX application (Figure 4, *p *< .05).

**Figure 5:**
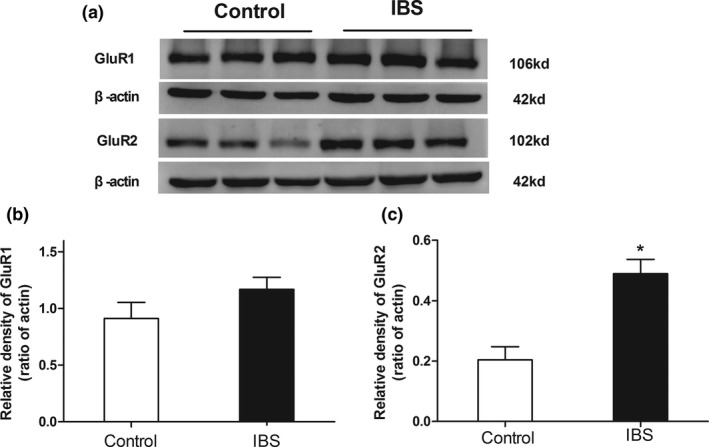
The expressions of hippocampal GluR1 and GluR2 in rats. (a) Typical Western blot results of hippocampal GluR1 and GluR2 in control and IBS‐like rats. (b) No significant difference was found between the GluR1 expressions of IBS‐like rats and those of control rats. (c) GluR2 expressions significantly increased in IBS‐like rats compared with control rats. *n *= 3, *: *p *<* *.05, vs. controls. IBS, irritable bowel syndrome

**Figure 6:**
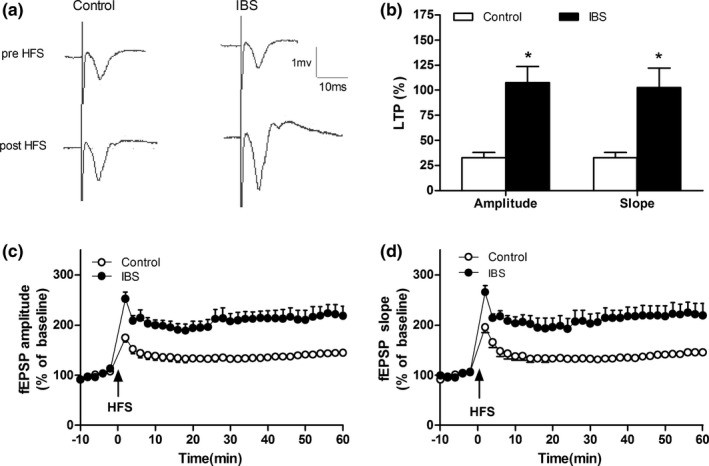
Hippocampal field potential before and after HFS in rats. (a) The original typical recordings of fEPSPs pre‐ HFS and 60 min after HFS in control and IBS‐like rats. (b) The statistical chart of the amplitude and slope of LTP of hippocampal slices 60 min after HFS in rats. (c) The standardized fEPSPs amplitude in rats. (d) The standardized fEPSPs slope in rats. *n *= 6, *: *p *<* *.05, vs. controls. HFS, high‐frequency stimulation; IBS, irritable bowel syndrome; fEPSP, field excitatory postsynaptic potential; LTP, long‐term potential

**Figure 7:**
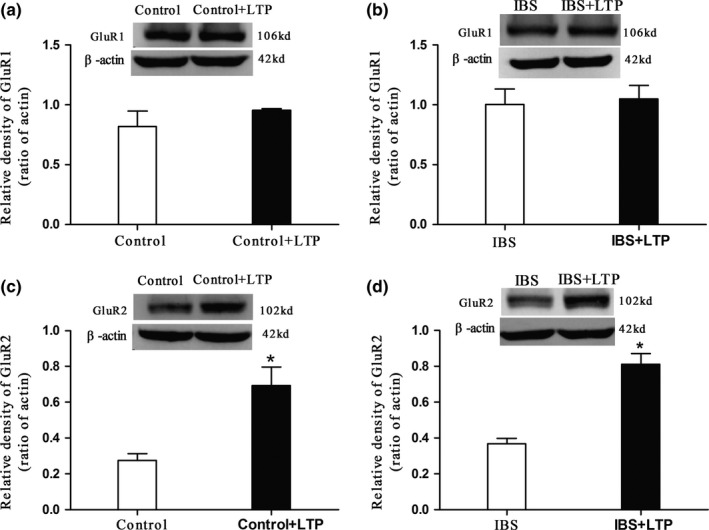
Hippocampal GluR1 and GluR2 expressions before and 60 min after HFS. (a, b) Typical original Western blot results of GluR1 in hippocampal slices. No significant change was found between the hippocampal GluR1 expressions before and 60 min after HFS in control and IBS‐like rats. (c, d) Typical original Western blot results of GluR2 in hippocampal slices before and 60 min after HFS. Hippocampal GluR2 expressions increased significantly 60 min after HFS in control and IBS‐like rats. LTP was induced by HFS and recorded 60 min after HFS. *n *= 3. *: *p *<* *.05, vs. pre‐HFS. HFS, high‐frequency stimulation. IBS, irritable bowel syndrome; LTP, long‐term potential

The expressions of hippocampal GluR2 (Figure 5, *p *< .05), not GluR1 (Figure 5) were significantly higher in IBS‐like rats when compared with controls. LTP, induced by HFS and recorded 60 min after HFS at SC‐CA1 synapses, was significantly increased in IBS‐like rats when compared with controls (Figure 6, *p *< .05). The expressions of hippocampal GluR2, but not GluR1, significantly increased in both control and IBS‐like rats 60 min after HFS (Figure 7, *p *< .05).

## 4 Discussion

In the present study, we found that hippocampal GluR2 may contribute to the enhanced LTP and induce visceral hypersensitivity in IBS‐like rats. Firstly, in IBS‐like rats, intrahippocampal injections of CNQX alleviated the visceral hyperalgesia. Secondly, the expressions of hippocampal GluR2 increased significantly. Thirdly, LTP induced by HFS at SC‐CA1 synapses was significantly enhanced. Finally, hippocampal GluR2 expressions up‐regulated significantly in control and IBS‐like rats 60 min after HFS.

### 4.1 The effect of AMPARs on IBS‐like rats

AMPARs are important ionotropic glutamate receptors, mediating most fast excited nervous transmissions. They participate both in physiological and pathophysiological sensations (Triller & Choquet, 2005). In our study, hippocampal injection of high dose CNQX (an AMPARs antagonist) could significantly attenuate visceral hypersensitivity in rats, and the inhibitory rates of IBS‐like rats were higher than that of control rats. Medial dose CNQX only showed alleviatory effects in IBS‐like rats but not in controls. Our results are in accordance with those of Lin (Lin & Al‐Chaer, 2005): low, medial, and high doses of CNQX all significantly decreased the neuronal responses to CRD in IBS‐like rats. However, medial and high doses of CNQX decreased the neuronal responses to CRD in controls. Wu et al. (2008) claimed AMPARs were responsible for anterior cingulate cortex (ACC) nociceptive transmissions in control rats. These results suggest that AMPARs play a more important role not only in a visceral physiological sense but also in the formation of chronic visceral hyperalgesia. Other scholars pointed out that APMARs in primary somatosensory cortex selectively mediate chronic inflammatory pain, but not acute or physiological pain (Eto et al., 2011). Injection of CNQX in primary somatosensory cortex significantly reduced ACC field potentials and increase pain thresholds in Complete Freund's adjuvant (CFA) induced inflammation pain mice, but had no effect in control mice (Eto et al., 2011). Intrathecal administration of AMPARs antagonist show dose‐dependent analgesia effect in acute pain assessed by tail‐flick (Advokat & Rutherford, 1995) or hot plate (Nishiyama, Yaksh, & Weber, 1998). Injection of AMPARs antagonist in amygdala did not change pain reactions in acute pain rats (Ghalandari‐Shamami, Hassanpour‐Ezatti, & Haghparast, 2011). Therefore, the roles of AMPARs in different pains are not always the same.

The analgesia effect of high dose CNQX in IBS‐like rats reach a peak at 30 min after injection, then decreased gradually, lasted about 90 min, and pain reactions returned to normal 120 min after injection. There might be several reasons for this degeneration in analgesia effect. First, brain CNQX concentration decreased as time went on. Second, pain perceptions from other brain areas exceeded the inhibition of CNQX. Third, CNQX only selectively blocks AMPARs, the functions of other glutamate receptors still exist. Fourth, visceral pain sensation may be recovered through other mechanisms apart from AMPARs. Park announced an intrathecal administration of CNQX could significantly alleviate the CFA‐induced mechanical pain and thermal pain up to 24 hr (Park et al., 2008). A plausible reason for the time difference might be that our study was of visceral pain, and theirs was of somatic pain. The analgesia effects of CNQX on different types of pain might be different.

GluR1 and GluR2 are considered to be closely related to pain sensation among the AMPAR subunits. Choi and Pezet have revealed that GluR1 expression in the spinal dorsal horn neuron membrane up‐regulated formalin‐induced inflammation pain in animals, but the expression of GluR2 was unchanged (Choi, Svensson, Koehrn, Bhuskute, & Sorkin, 2010; Pezet et al., 2008). Park et al. (2008) observed GluR1 and GluR2 changed in CFA‐induced chronic pain animal models, i.e., membrane GluR1 increased, and GluR2 decreased. With neuropathic pain, Chen, Zhou, Byun, & Pan (2013) found the spinal dorsal horn neuron membrane GluR2 decreased, and the cytoplasm GluR2 increased while GluR1 had no significant changes. It is obvious that analgesia mechanisms of AMPARs vary in different types of somatic pains. Zhou, Huang, Gao, Zhang, & Jiang (2014) observed GluR2 up‐regulated in ACC of visceral pain model rats and inferred it might be the mechanism of visceral hyperalgesia and synapse plasticity. In this study, hippocampus GluR2 expressions were significantly elevated in IBS‐like rats, which is in accordance with Zhou's opinion that GluR2 might play an important role in visceral hyperalgesia formation. The AMPARs‐mediated excitatory glutamate synapse transmissions were critical participants in the neuron development, synapse plasticity, and remodeling. NMS may lead to AMPARs’ expression change, thus affecting glutamate transmission (Katsouli et al., 2014). Thus, we deduce NMS stress caused AMPARs expression change in rats, which affected neuron growth and synapse plasticity, generating visceral hyperalgesia in adult rats.

### 4.2 The effects of AMPARs in synapse plasticity

AMPARs regulate noxious and non‐noxious sensation transmission in the spinal cord. They participate in central sensitization of different pains. Li et al. (2010) considered chronic pain as a typical example of central sensitization. Central sensitization is a persistent plastic change of synapse transmission developed during or after noxious stimulations. LTP is one kind of synapse plasticity. The different subunits of AMPARs play important roles in LTP. Mahanty and Sah detected that calcium‐permeable AMPA receptors mediate LTP in interneurons in the amygdala (Mahanty & Sah, 1998). Also, Plant et al. (2006) observed the transient incorporation of native GluR2‐lacking AMPA receptors during hippocampal LTP. However, Gray, Fink, Sarinana, Vissel, & O'Dell (2007) demonstrated that LTP in the hippocampal CA1 region does not require insertion and activation of GluR2‐lacking AMPA receptors. In this study, GluR2 expression increased after high‐frequency stimulation in the hippocampus slides of IBS‐like rats, which was in accordance with the previous studies, indicating that GluR2 plays an important role in LTP maintenance and synapse plasticity in IBS‐like rats.

### 4.3 Hippocampus LTP, memory, and pain

It is well known that the hippocampus is involved in many functions of the brain, such as learning and memory, mood and emotion, feelings and motivation.However, there are always controversies concerning whether the hippocampus participates in pain procession. Shih reported formalin‐induced nociceptive processing increased blood oxygenation level‐dependent signals in the hippocampus (Shih et al., 2008). The reorganizations of signal processing in the hippocampus and between hippocampus and cortex seem to contribute to the transition from subacute to chronic pain (Mutso et al., 2014). Pain behaviors were significantly reduced by the injection of NMDA receptor antagonists in the hippocampus (Soleimannejad, Naghdi, Semnanian, Fathollahi, & Kazemnejad, 2007). Our results showed that the hippocampus AMPA receptor antagonist administration significantly alleviated the visceral pain in IBS‐like rats. These results suggest that the hippocampus is involved in pain‐related processing.

A great number of studies have proved that sensory‐related long‐term synaptic plasticity is a crucial mechanism of chronic pain (Woolf & Salter, 2000; Zhuo, 2008). Wei, Xu, Qu, Milbrandt, & Zhuo (2000) first recorded a hippocampal reaction to peripheral noxious stimulations in adult rats. Zhao reported that in a bee venom‐induced persistent pain animal model, hippocampus LTP was remarkably enhanced (Zhao et al., 2009). In this study, we observed that LTP induced by HFS in the hippocampus CA1 region significantly increased in IBS‐like rats, which is in accordance with our previous data (Chen et al., 2014). Our data suggest that the hippocampus LTP might be the cellular mode of pain. However, further investigations are still needed to provide direct evidence for the relationship that exist between hippocampus LTP and pain.

The formation of chronic pain is closely similar to those of learning and memory. Moreover, chronic pain can lead to various changes in the nervous system like those in memory process and these changes last after the cure of the injuries. From functional modification to structural recombination, these different types of memories were also occurring in chronic visceral pain (Yi & Zhang, 2011). Central sensitization has long been considered as a critical mechanism in chronic pain, and LTP is the cellular basis of memory. Ji found astonishing similarities between central sensitization and LTP after comparing the production and sustaining of them, particularly in the regulations and transportations of NMDARs and AMPARs (Ji, Kohno, Moore, & Woolf, 2003). Among the over 100 kinds of LTP‐related factors, many of them participate in spinal central sensitization and lead to hyperalgesia. From this, we can deduce that there might be similar molecular mechanisms underlying chronic pain and memory. Chronic pain could be called a kind of noxious memory, but more researches should be carried out to understand and distinguish the roles of hippocampal LTP in pain and memory.

## Conflict of Interest

None declared.
